# Red and green defocus curves and duochrome test in different age groups

**DOI:** 10.1016/j.optom.2023.100497

**Published:** 2023-12-20

**Authors:** Riccardo Rolandi, Fabrizio Zeri, Alessandro Duse, Giulia Carlotta Rizzo, Erika Ponzini, Silvia Tavazzi

**Affiliations:** a Department of Materials Science, University of Milano-Bicocca, Milan, Italy; b Research Centre in Optics and Optometry (COMiB), University of Milano-Bicocca, Milan, Italy; c College of Health and Life Sciences, Aston University, Birmingham, UK

**Keywords:** Duochrome, Defocus curve, Eye examination, Age, Visual acuity

## Abstract

**Purpose:**

To compare the eye defocus curves (DCs) obtained with stimuli on red, green, and white backgrounds and to investigate the applicability of the duochrome test (DT) in different age groups.

**Methods:**

12 elderly (ELD: 59.3 ± 3.9 years) and 8 young (YG: 22.1 ± 1.1 years) subjects were recruited. An optometric assessment with the DT was carried out to obtain the subjective refraction at distance. DCs at distance on green, white, and red backgrounds were measured and the following parameters were deduced: dioptric difference between red-green, green-white, red-white focal positions (minima of the DCs), best corrected visual acuity (BCVA), and widths of the DCs for red, green, and white.

**Results:**

The DC difference between the green-white focal positions (mean ± standard deviation) was -0.12±0.17 diopters (D) (ELD, *p* *=* 0.012) and -0.11±0.12 D (YG, *p* *=* 0.039), while the red-white difference was not statistically significant. The DC red-green difference was 0.20±0.16 D (ELD, *p* *=* 0.002) and 0.18±0.18 D (YG, *p* = 0.008). The ELD BCVA with green background was significantly worse than BCVA with red (*p* = 0.007) and white (*p* = 0.007). The mean value of the DC's width in ELD for green (1.01±0.36 D) was higher than for red (0.77±0.21 D) and for white (0.84±0.35 D), but with no statistical significance.

**Conclusion:**

Both age groups showed a slight focusing preference for red when using white light. Moreover, ELD showed a worse BCVA with a green compared to a red background. Despite these results deduced by DC analyses, these aspects do not compromise the possibility of using the DT in clinical practice both in the young and in the elderly. Furthermore, the difference of about 0.20 D between red-green DC in both groups confirms the clinical appropriateness of the widespread use of 0.25 D step as the standard minimum difference in power between correcting lenses.

## Introduction

Since its clinical introduction in 1927 by Brown,^1^ eye care professionals have been using the duochrome test (also known as bichromatic or dichromatic test)^2^ usually at the end of monocular/binocular refraction procedure^3^^,^^4^ to adjust the spherical component of the correction and to reach the subjective end-point of distance refraction. This test exploits the presence in the eye of longitudinal, or axial, chromatic aberration (LCA) (approximately 2 diopters between 400 and 700 nm)^4^ that provides different focal points for different wavelengths due to the relationship between the refractive index of the ocular media and wavelength. The duochrome test makes use of a typical distance visual chart split in two backgrounds, red and green (of approximately 620 nm and 535 nm respectively, according to the British Standard 3668^2^), in which the same black stimuli are presented. Using these two different wavelengths, two different focal points are created that are expected to lie before and after the optimal focal point that, in the case of emmetropia, is expected on the retina. In an emmetropic eye, this optimal focal point placed on the retina is expected to correspond to a wavelength of approximately 570 nm (yellow light^2^^,^^5^). In the case of ametropia the optimal focal point is not on the retina, therefore, one of the two focal points, generated by the LCA, will be closer to the retina. This will increase the contrast of the stimuli on the background with the wavelength closer to the retina (red for myopia, green for hyperopia). The duochrome test is used during the refining of the spherical component of refraction and it is performed by asking the patient to indicate when letters appear equally clear on both backgrounds.

Considering the rationale of the duochrome test, changes in LCA across ages might affect its reliability. In the literature, LCA is typically measured in a broader range of wavelengths, usually from 450 nm to 650 nm, compared to those of the duochrome test. Moreover, literature on this topic shows conflicting results. For example, a study on fifty-eight subjects of different ages found a reduction in ocular LCA by a factor of four from 10 to 40 years to 70–80 years.^6^ The same authors also concluded that most of the reduction in LCA would occur at the onset of presbyopia.^6^ Another study,^7^ with only seven subjects, supported the LCA reduction with age. The reduction in LCA reported in this latter study, however, mainly concerned short wavelengths below 550 nm. On the contrary, longitudinal results reported by Ware^8^ regarding two subjects from 40 to 65 and from 30 to 55 years, respectively, showed essentially no change in ocular LCA in the 420–740 nm range of wavelengths. More recently, Tanaka et al.^9^ compared color visual acuity (VA) measured by Landolt rings with dominant-wavelengths equal to 607, 566, 488, and 440 nm on a white background in the age range 27–47 years. These authors concluded that a significant positive correlation occurs between LCA and age. However, this and other studies on this topic are cross-sectional studies and with investigation techniques that required subjects to assess clarity of a target to measure ocular LCA. This approach rests on the assumption that subject's ability to assess precision of focus does not vary with age or wavelengths.^10^^,^^11^ However, Howarth et al.^12^ speculated that smaller pupils and reduced transmission at the shortest wavelengths in older subjects can lead to illusory decline in ocular chromatic aberration and concluded that LCA is independent of age: LCA drop would be determined by ocular media scattering rather than refractive index changes.

The purpose of the present study is to compare the dioptric focal positions and the best visual acuities inferred from red, green, and white light defocus curves (DCs) with each other and with the results of the duochrome test and to investigate the applicability and relevance of the duochrome test in different age groups.

## Materials and methods

### Participants’ recruitment

All procedures were in accordance with the Declaration of Helsinki and approved by the Board of Optics and Optometry of the host University (November 2, 2020, n° 10/2020). Written informed consent was obtained from all subjects prior to participation in the study.

Every subject underwent the first phase of the study consisting of an optometric assessment and a slit lamp examination (paragraph 2.3) in order to verify the inclusion criteria and to determine the refraction for far distance, needed as baseline for the defocus curve (DC) measurements (paragraph 2.4). Subjects were recruited according to the following inclusion criteria and clustered in two different groups:

▪ not having current or previous known ocular and systemic pathologies;
▪ having clear ocular media;
▪ not being under medical ocular and systemic therapy;
▪ having ametropia (sphere) in each eye in the range from −6.50 to + 2.50 diopters (including range limits) with astigmatism between 0 and −1.50 diopters;
▪ having a monocular best corrected visual acuity (BCVA) of 0.40 or better measured in logarithm of the minimum angle of resolution (logMAR);
▪ not being pseudophakic;
▪ aged less than 40 years (allocated in the young group: YG) or in the range between 55 and 70 years (allocated in the elderly group: ELD).

The number of subjects who met the inclusion criteria was 34 (15 in the ELD group and 19 in the YG group). Some subjects were excluded retrospectively from the data analysis as described in paragraph 2.4. The final resulting number of subjects was 20 (12 in ELD group and 8 in YG group).

### Illuminance measurements

**Fig. 1:**
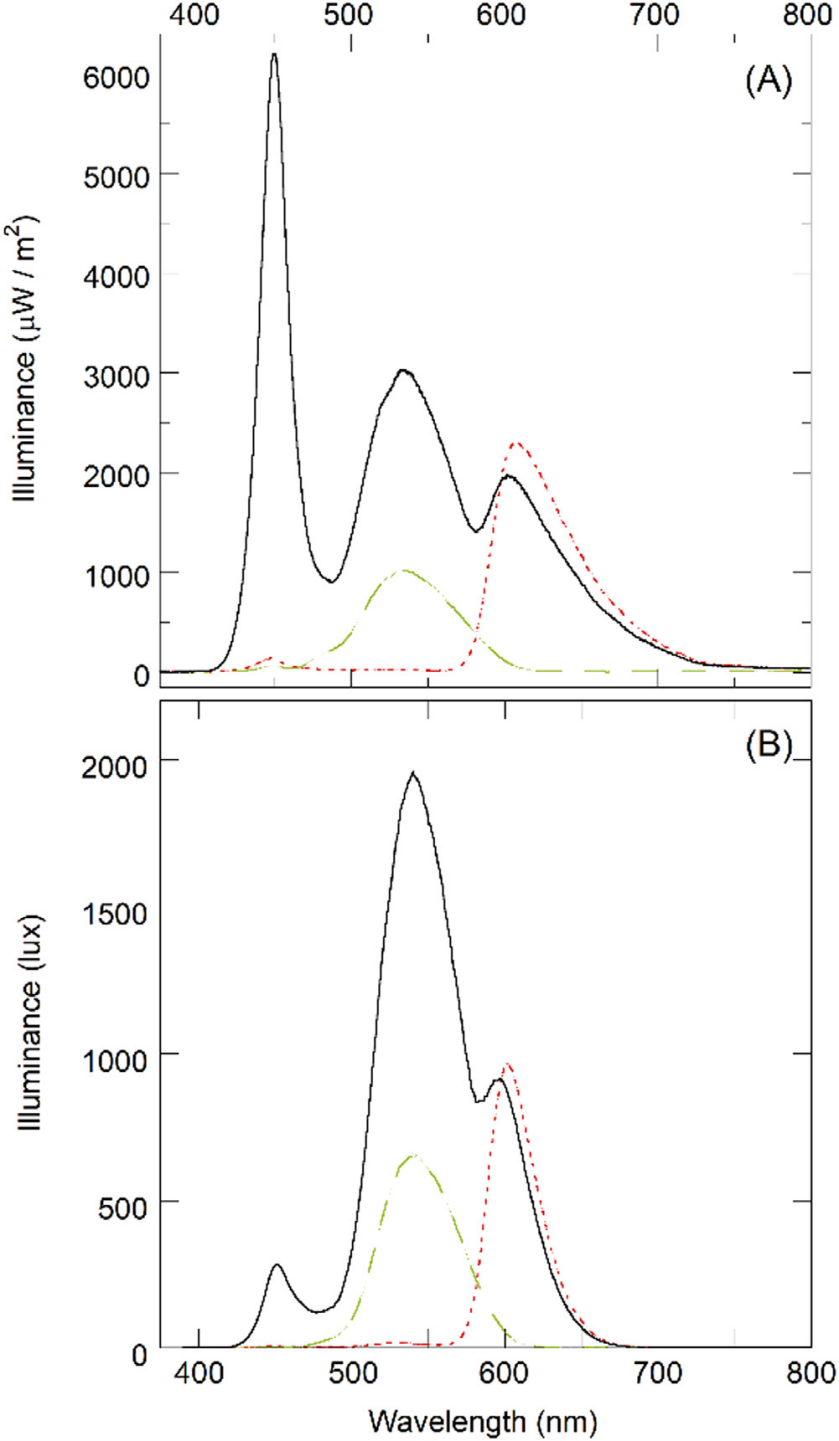
Illuminance spectra (power per unit illuminated area) of the red (dotted line), green (dashed line), and white (continuous line) backgrounds measured on an illuminated surface parallel to the LCD display at a distance of 11 ± 1 cm either (A) in µW/m^2^ or (B) in lux (lumen/m^2^).

The illumination in the room (in the position of the subject examined) was measured by a HT307 luxmeter (HT Italia, Faenza, Italy) and was found to be 8 ± 2 lux. The emission spectrum of the display used for the visual analyses (LCD LED Vision Chart, CSO, Florence, Italy) was measured by a Hamamatsu C10082CAH spectrophotometer (Hamamatsu Photonics, Japan) combined with a power meter Ophir Nova P/N7Z01500 (Ophir Optronics, Jerusalem), for white, red, and green backgrounds. The illuminated surface of the power meter was arbitrarily placed 11 ± 1 cm away from the LCD display and parallel to it. The illuminance spectra were measured in W/m^2^ units (Fig. 1A).

Three peaks were identified in the case of the white background at 450±5 nm, 535±5 nm, and 610±5 nm. Only the second and third peaks were found in the case of green and red backgrounds, respectively. The 1931 CIE curve was then adopted for the conversion from W/m² to lux units (spectra in lux are shown in Fig. 1B). The integrated values underlying the curves between 380 and 780 nm were found to be 39,700±1000 lux (red), 40,800±1000 lux (green), and 160,000±3000 lux (white). These values refer to the distance of 11±1 cm from the display and the absolute values have little significance for optometric measurements at far distance. For this reason, the intensity (measurement angle 1°) per unit of emitting area (luminance) was also measured with a CS2000 spectroradiometer (Konica Minolta, Japan). Luminance was found to be 43±1 cd/m^2^ (for red), 49±1 cd/m^2^ (for green), and 208±2 cd/m^2^ (for white). The same instrument also provides the chromatic coordinates (x,y) in CIE 1931 for red (0.6487;0.3357), green (0.2994;0.6092), and white (0.3067;0.3362). The respective values in a sRGB 8-bit system are (255;7;0), (0;217;0), and (255;255;255) for red, green, and white, respectively.

### Subjective examination

A preliminary objective refraction measurement was obtained using a Canon RK – F1 Auto Refractometer (Canon Inc., Tokyo, Japan) under the abovementioned lighting conditions (paragraph 2.2). The habitual prescription, if present, or the objective refraction was placed in a phoropter as starting point to perform a non-cycloplegic subjective refraction at far distance (4.30 m) with visual stimuli presented on the LCD Vision Chart (CSO, Florence, Italy) described in section 2.2 using white light at maximum contrast. Once reached the sphero-cylindrical refraction with the maximum plus to maximum visual acuity (MPMVA) criterion,^13^ the duochrome test was monocularly presented on the same LCD display used during subjective refraction procedure. The best subjective refraction (BSR) was obtained when the subjects reported equal clarity (when possible) or the least difference for letters on red and green backgrounds with the duochrome test. The final duochrome end-point sphero-cylindrical lens was collected for each subject and the spherical equivalent (SE) was calculated by adding half of the cylinder power to the sphere power. With the BSR arranged in a trial frame, the high-contrast BCVA was measured presenting a row of five Sloan letters, displayed on the LCD described above, using a descending logarithm progression of 0.10 logMAR. A forced choice procedure was used in which subjects were asked to guess when the size of the letters was close to the VA threshold and uncertainty was present, until five errors for a row were counted^14^ with a letter-by-letter (0.02 logMAR/letter) scoring criterion to assess the threshold. Every single 5-letter row was randomly generated among 28 different sets of letters with balanced readability and presented in isolation.^15^

### Defocus curves (DCs)

The aim of this phase of the study was to determine the degradation of the VA due to additional spherical dioptric power compared to BSR for three different backgrounds: white, red, and green (see paragraph 2.2 for illuminance spectra). In this phase, for each subject, only the eye with the best BCVA, as found in the preliminary assessment, was examined. Spherical lenses, ranging from −1.50 to +1.50 diopters with steps of 0.50^16^ were randomly added monocularly in front at the BSR inserted in a standard trial frame. VA was measured to achieve the full DC, using black Landolt rings (minimum luminance). Five rings (randomly oriented in eight directions) were presented isolated for each angular size and the number of errors was recorded using a forced choice procedure (subjects were asked to guess when the size of the rings was close to the VA threshold and uncertainty was present). Starting from 0.40 logMAR, a descending logarithm progression of 0.10 logMAR was adopted, until five mistakes on the same line were reached.^14^ A letter-by-letter (0.02 logMAR/letter) scoring criterion was used to assess the threshold.

**Fig. 2:**
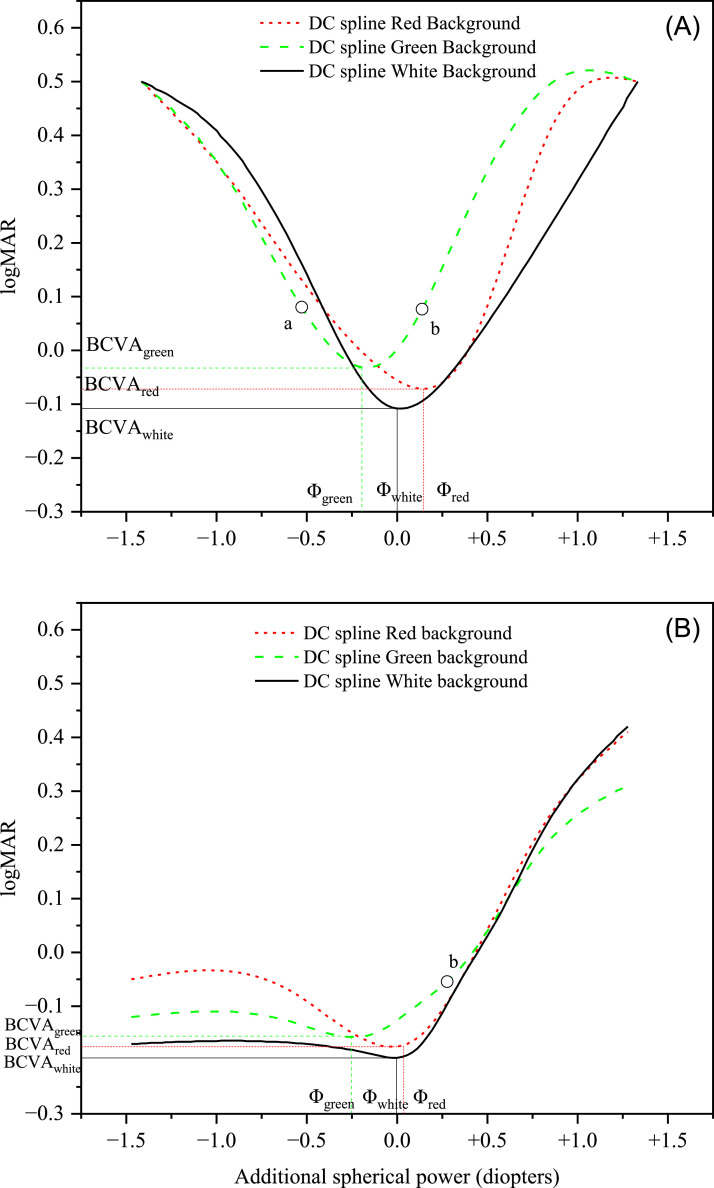
DCs with red, green, and white backgrounds of one subject of the ELD group (Panel A) and one subject of the YG group (Panel B) taken as examples. Points marked as “a” and “b” on the green DC in panel A and point marked as “b” in panel B were used to calculate the DC width (W_green_) as described in paragraph 2.4.

For each subject and for each additional lens, the actual additional spherical power affecting the ocular system was determined taking into account the BSR and the distance between the ocular apex and the frame. The data were then used to find the DC by regression of the experimental logMAR values with a cubic spline function. After getting the DC through data regression, the dioptric positions of the minima (BCVA) for red and green light were compared to the position of the minimum (BCVA) with white background. For this purpose, the dioptric position (abscissa values) of the three DCs were all translated by the same amount for each subject to set at zero the dioptric position of the minimum of the subject's DC in the case of white light, thus considering the white as reference. The positions of the DC minima after this shift are here called Φ_green_ and Φ_red_ for green and red background, respectively (the position for white light is Φ_white_ = 0 for every subject). With this procedure, a negative relative dioptric value corresponds to myopic defocus and a positive dioptric value corresponds to hypermetropic defocus. The amount of this value is the dioptric distance between the BCVA lens for the specific background with respect to the BCVA lens in the case of the white background (reference). Three DCs obtained for one subject of the ELD group and one subject of the YG group are shown in Fig. 2A and 2B, respectively, as examples. The spherical power of the prescription lens (BSR) obtained with the duochrome test in the preliminary examination was also translated by the same amount as for the three DCs, thus obtaining the value Φ_duo_. In summary, the following data were deduced for each subject:

▪ the coordinates (Φ_green_; BCVA_green_) of the minimum of the DC with green background;
▪ the coordinates (Φ_red_; BCVA_red_) of the minimum of the DC with red background;
▪ the difference LCA_RG_ = Φ_red_ - Φ_green_;
▪ the average between Φ_green_ and Φ_red_ (Φ_meanRG_);
▪ the ordinate value (BCVA_white_) of the minimum of the DC with white background, the abscissa value being set at zero for each subject;
▪ the relative spherical power (Φ_duo_) corresponding to the prescription lens (BSR) obtained with the duochrome test in the preliminary examination (paragraph 2.3) with respect to the abscissa of the DC minimum with white light; similarly, as Φ_green_, Φ_red_, and Φ_white_, also for Φ_duo_ the actual spherical power affecting the ocular system was determined taking into account the BSR and the distance between the ocular apex and the lens;
▪ the widths of all three DCs (W_red_, W_green_, W_white_) calculated as the difference between the two abscissas corresponding to visual acuity 0.1 logMAR^17^ greater than the BCVA (indicated for example by the points a and b in one of the DCs in Fig. 2A). Concerning the YG group, due to accommodation the DCs were typically not symmetrical and the width was calculated as twice the difference between the abscissa of the DC minimum and the abscissa of point b (Fig. 2B).

To avoid uncertain data, subjects were retrospectively excluded from this study if the minimum could not be unambiguously determined even in only one DC out of three. In particular, 3 out of 15 ELD and 11 out of 19 YG participants were excluded due to unreasonable fluctuations in at least one of their three DCs, caused by dubious answers during the analyses, and/or to difficulty in finding a clear DC minimum.

### Statistical analysis

Demographic and optometric information are summarized in Table 1. Non-parametric statistics were used with statistical significance set to 0.05. For paired and unpaired comparisons, Wilcoxon signed-rank and Mann-Whitney U test were used, respectively. When testing multiple hypotheses, the Bonferroni correction was adopted and each individual hypothesis was tested at a significance level equal to α/m, where α is the desired overall significance level (α=0.05) and m is the number of hypotheses. All statistical analyses were performed using OriginPro, Version 2022b (OriginLab Corporation, Northampton, MA, USA).

**Table 1:** Demographic and optometric information of the eyes/subjects of the study. Mean, standard deviation (SD), minimum (Min), and maximum (Max) values both for ELD and YG group are reported (N: number of subjects; N_male_: number of males).

	Group							
	ELD				YG			
N (N_male_)	12 (6)				8 (3)			
	Mean	SD	Min	Max	Mean	SD	Min	Max
Age (years)	59.3	3.90	55	66	22.1	1.10	21	24
Sph (D)	−2.54	2.17	−6.25	0.75	−0.69	1.79	−3.25	2.25
Cyl (D)	−0.58	0.46	−1.25	0.00	−0.38	0.42	−1.00	0.00
SE (D)	−2.83	2.06	−6.25	0.50	−0.87	1.66	−3.25	1.75
BCVA	−0.06	0.12	−0.21	0.17	−0.15	0.08	−0.25	−0.04

Based on a preliminary evaluation of the expected results, a threshold value for the difference between the means that can be proved statistically with significance level at 0.05/m with twenty participants was calculated. For the relative dioptric values (results shown in Table 2, as discussed in the following section), the threshold was found to be 0.12 diopters (assuming 0.13 diopters as standard deviation of the results). For the BCVA (results shown in Table 3) the threshold was found to be 0.12 logMAR (assuming 0.12 logMAR as standard deviation). For the DC width (results shown in Table 4) the threshold was found to be 0.31 diopters (assuming 0.3 as standard deviation).

**Table 2:** Mean, standard deviation (SD), minimum (Min), and maximum (Max) of Φ_green_, Φ_red_, Φ_duo_, Φ_meanRG_, and LCA_RG_ (Φ_white_ = 0 for all subjects, all values in diopters) for both age groups (ELD and YG) and p-values of the statistical comparison among them (ELD vs YG, last column on the right). Brackets with p-values for paired comparisons: left side for ELD group, right side for YG group. P-values are indicated in bold when below the threshold of statistical significance, taking into account the Bonferroni correction.

**Table 3:** Mean, standard deviation (SD), minimum (Min), and maximum (Max) of the BCVA values (in logMAR) with the three backgrounds for both age groups (ELD and YG) and p-values of the statistical comparison among them (ELD vs YG, last column on the right). Brackets with p-values for paired comparisons: left side for ELD group, right side for YG group. P-values are indicated in bold when below the threshold of statistical significance taking into account the Bonferroni correction.

**Table 4:** Mean, standard deviation (SD), minimum (Min), and maximum (Max) of the width (in diopters) with the three backgrounds for both age groups (ELD and YG) and p-values of the statistical comparison among them (ELD vs YG, last column on the right). Brackets with p-values for paired comparisons: left side for ELD group and right side for YG group. P-values are indicated in bold when below the threshold of statistical significance taking into account the Bonferroni correction.

## Results

Table 2 shows the mean values of Φ_red_ and Φ_green_ calculated on the ELD and YG subjects (Φ_white_ is zero because the DCs of every subject were translated to set at zero the minimum of the DC taken with white background). As expected, mean data show positive dioptric values for red focal position (Φ_red_) and negative dioptric values for green focal position (Φ_green_) relative to white reference, with green-white mean dioptric distance (± standard deviation) of −0.12±0.17 (ELD) and −0.11±0.12 diopters (YG), and red-white mean dioptric distance of 0.08±0.13 (ELD) and 0.08±0.11 diopters (YG). As can be seen, the difference in absolute value with respect to white is slightly higher for green. Indeed, Φ_green_ reached statistically significant difference with respect to the zero-reference value for white (ELD *p* *=* 0.012; YG *p* *=* 0.039). This was not the case for Φ_red_ for both groups, although the threshold of significance was almost reached (ELD *p* *=* 0.064; YG *p* *=* 0.078). As expected, a clear difference (ELD *p* *=* 0.002; YG *p* = 0.008) was found between the red and green chromatic positions. In this respect, the mean LCA_RG_ (± standard deviation) was 0.20±0.16 and 0.18±0.18 diopters for ELD and YG group, respectively. No significant differences were found (ELD *p* = 0.622; YG *p* = 0.945) when comparing Φ_duo_ obtained with the duochrome test (ELD −0.02±0.19; YG 0.00±0.16 diopters) with the reference value Φ_white_. The values of Φ_meanRG_ are −0.02±0.13 and −0.01±0.07 diopters for ELD and YG groups, respectively. Also in this case, no difference was found between Φ_meanRG_ and Φ_white_ (ELD *p* = 1.000; YG *p* *=* 0.641). Other p-values for the paired comparisons between Φ_red_, Φ_green_, Φ_white_, Φ_duo_, and Φ_meanRG_ are reported in Table 2 (left and right sides). Concerning the direct comparison between the two age groups, no differences in the dioptric powers were found between ELD and YG (p values are reported in the last column of Table 2 for each dioptric variable).

As far as BCVA is concerned (Table 3), the data show a peculiarity for the ELD group in the specific case of green. BCVA for ELD group was +0.03±0.14 and −0.02±0.11 logMAR for green and red, respectively, the two values being statistically different (*p* = 0.007). Not only compared to red, but also compared to white, for the ELD group BCVA_green_ was found to be significantly worse (*p* = 0.007) than BCVA_white_ (BCVA_white_ = −0.06±0.12 logMAR). On the contrary, no difference was found between ELD BCVA_red_ and BCVA_white_ (*p* = 0.064). The scenario was different in the case of YG subjects. Indeed, the difference between BCVA_green_, BCVA_red_, and BCVA_white_ was no more observed (−0.13±0.08, −0.13±0.11, and −0.15±0.08 logMAR for green, red, and white, respectively, with *p*>0.05 for all comparisons). The previous considerations concern the comparison between the three BCVAs in subjects belonging to the same age range. When comparing the two age groups, results from the YG group clearly showed lower logMAR values (better BCVA) compared to ELD using green background (BCVA_green_ *p* = 0.004). Concerning white light (Table 3), the BCVA was still better in YG than ELD, but without reaching the statistical significance level (BCVA_white_ *p* = 0.098). In general, the greatest BCVA difference between YG and ELD was found for green and the mean value of BCVA_green_ in logMAR for ELD was the only one that was greater than zero (Table 3). Finally, when comparing DCs widths (Table 4), the mean value of the DC width in ELD for green (1.01±0.36 D) was higher than for red (0.77±0.21 D) and for white (0.84±0.35 D), but with no statistical significance. No significant differences were found between backgrounds and between age groups.

## Discussion

A first conclusion drawn from the present study is that the LCA_RG_ deduced from the DCs does not decrease with age (21–24 vs 55–66 years) in the spectral range between 535 and 610 nm. Indeed, the dioptric difference (LCA_RG_) between the two colors used was found to be 0.20±0.16 diopters for the ELD group and 0.18±0.18 for the YG group. These LCA_RG_ results are consistent with some results reported in the literature.^6^, ^7^, ^8^^,^^12^^,^^18^ For example, Millodot^6^ reported that LCA depends on age, but the majority of the decrease was for wavelengths below about 540 nm, outside the spectral range of interest of the duochrome test.

Although equal extent of LCA_RG_ in the two age groups was found, differences cannot be excluded between ELD and YG concerning pupil size and higher order aberrations (HOAs) of the eye.^18^, ^19^, ^20^ However, these variables were not measured in this work. Conversely, experimental evidence of differences is here reported as a function of age in BCVA, especially in the case of green background. The observed worse BCVA in the elderly at shorter wavelengths (green compared to red) can be tentatively attributed to the increase of wavelength-dependent intraocular scattering.^12^ In turn, worse VA with green background compared to red could be the origin of the higher DC widths with green background (Table 4). In other words, a worse VA could make the eye more tolerant with respect to changes of the dioptric power from the optimal optical prescription. Even though it is not mentioned in the results section, the Spearman's rank correlation coefficient (rs) was computed to assess the relationship between BCVA_green_ and W_green_ in the ELD group and, indeed, a significative good correlation was found (rs = 0.67, *p* = 0.017). In summary, despite the worse BCVA with green background compared to red in the ELD people and despite their general worse BCVA compared to YG subjects, a similar LCA_RG_ was found for the two age groups.

The results of the present study also show that the BCVA found with white background leaves subjects with a slight preferred focus towards the red that is independent from age. On the basis of the mean values of Φ_green_ and Φ_red_ shown in Table 2, it is possible to evaluate approximately which wavelength corresponds to Φ_white_. For this purpose, through a simple model consisting of a single transparent medium and considering that the power of the eye is proportional to its refractive index, it is possible to assume that the relative dioptric power depends on wavelength as

(1) $$\Phi \left(\lambda \right)=\text{A}+\frac{B}{\lambda^{2}}$$

where A and B are two constant values. This equation is in agreement with Cauchy's empirical model^21^ which describes the variation of the refractive index of transparent media as a function of the wavelength of the electromagnetic radiation.

**Fig. 3:**
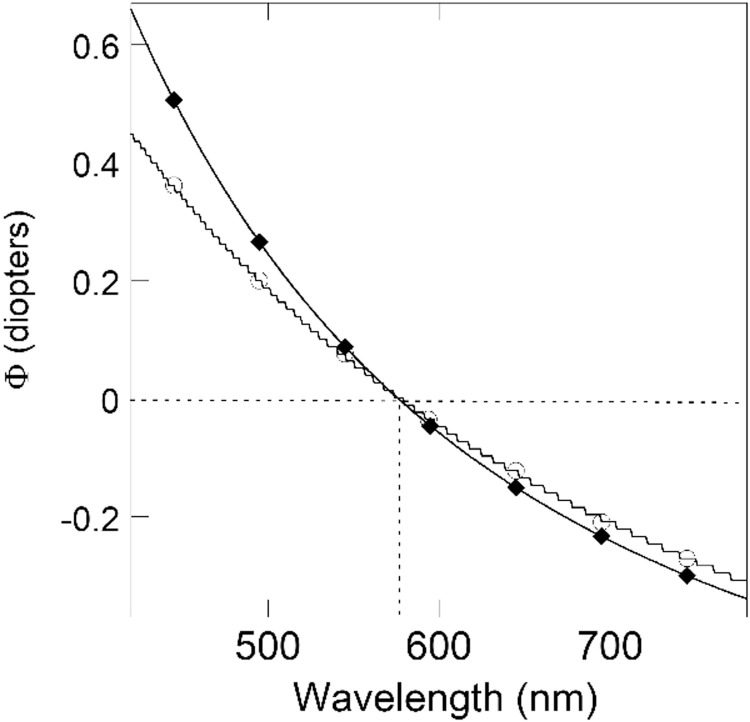
Relative dioptric power (with respect to the abscissa of the DC minimum with white light assumed at Φ_white_ = 0) either obtained by Eq. (1) with *A* = −0.74634, *B* = 247,970, and the wavelength in nanometers, on the basis of the mean Φ_green_ and Φ_red_ shown in Table 2 associated with the wavelengths 535 and 610 nm (line with diamonds) or obtained by the model mentioned by Thibos et al.^22^ for the refractive index of the eye. The horizontal and vertical dotted lines indicate the wavelength (576 nm) corresponding to Φ_white_ = 0.

The mean values of Φ_green_ and Φ_red_ shown in Table 2 associated with the wavelengths 535 and 610 nm respectively allow to deduce the parameters A and B and, in turn, the plot of Fig. 3 (line with diamonds). The dotted lines in Fig. 3 indicate the wavelength (576 nm) corresponding to Φ_white_ as deduced from Eq. (1). Since there are also models different from that of Cauchy^21^ to describe the dependence of the refractive index of transparent media on wavelength, another model mentioned by Thibos et al.^22^ was also adopted. The wavelength corresponding to Φ_white_ was substantially the same (line with empty circles).

The slight preference towards the red with white background partially confirms some findings reported in previous studies. For example, Sivak and Bobier reported that the emmetropic eye is in a red focal position when unaccommodated.^23^^,^^24^ They concluded that an optical correction based on the equal clarity of letters on red and green backgrounds is expected to produce an overcorrection (excess minus) for myopic eyes and an undercorrection (insufficient plus) in case of hyperopia. In the present work, the trend in favor of focusing of the red component with white light was found to be present but slight, with the consequence that the average of the measured dioptric values with red and green (Φ_meanRG_) was not statistically different from the power corresponding to the BCVA in white light (Φ_white_). Thus, the slight preference for red at far distance does not compromise the possibility of using the duochrome test. This is also confirmed by the fact that Φ_duo_ is not statistically different from Φ_meanRG_ and Φ_white_ (Table 2).

Finally, it is worth noting that, the difference of about 0.20 D between red-green DC found in the present study for both groups, confirms the clinical appropriateness of the widespread use of 0.25 D step as the standard minimum difference in power between correcting lenses.

## Conclusions

Experimental evidence at far distance is here reported of (i) a worse BCVA with green background in ELD subjects (55–66 years) compared to red background, (ii) a general worse BCVA of ELD compared to YG subjects, (iii) a slight focusing preference for red when using white light in both age groups. However, these differences do not compromise the possibility of using the duochrome test in clinical practice in the elderly. Indeed, a similar LCA_RG_ was found in ELD and YG groups and Φ_duo_ was not found to be statistically different from Φ_meanRG_ and Φ_white_ in both age groups.

## Conflicts of interest

None.
